# 

**Published:** 1919-12-06

**Authors:** 


### PASSING EVENTS AND TOPICS.

### Curious Clause in a Will.

Mr. N. E. Vouvalis made large bequests to the Greek Orthodox Community of Kalymnos for a hospital and schools. A clause in his will runs as follows: "My relatives are to make no complaint . . . because I do not leave them anything, as my principle is that those people who leave money to their relatives insult them by looking upon them as incapable people and turning them into idlers."

### A Patient's Gift to Longmore Hospital, Scotland.

The Annual Meeting of the Royal Hospital for Incurables was held in the Longmore Hospital, Edinburgh. Sir Henry Cook, who presided, gave the gratifying news that a patient, still in the hospital, had that day given a donation of £70 to the funds. Such an action proved the esteem in which the staff was held.

### Princess Mary at Stratford.

H.R.H. Princess Mary has promised to be present on Pound Day at Queen Mary's Hospital, Stratford, on December 11, at 2.30 p.m.

### Large Gift to Liverpool

The Liverpool Royal Infirmary has received from an anonymous donor the substantial gift of Twenty thousand pounds.

### Recent Official Publications.

(To be obtained from H.M. Stationery Office, Imperial House, Kingsway, W.C. 2. Prices include postage.)

Ministry of Health. General Housing Memorandum. 1½d. An Outline of the Practice of Preventive Medicine. 8½d.

Local Government Board. Reports and Papers on Malaria Contracted in England in 1918. 1s. 7½d. Statistics of the Incidence of Notifiable Infectious Diseases, 1918. 1s. 7½d.

Deaths from Starvation, or Accelerated by Privation. Return for the year 1918. 1½d.

Medical Research Committee. Special Reports: Studies of Influenza in Hospitals of the British Armies in France, 1918. 3s. 9d. Studies of Bacillary Dysentery Occurring in the British Forces in Macedonia. 3s. 2d. Wound Shock and Hæmorrhage. 4s. 5d.

Medical Research Committee. Special Reports: The Present State of Knowledge concerning Accessory Food Factors (Vitamines). 4s. 3d. Fifth Annual Report, 1918-19. 8d.

Local Government Board. Forty-eighth Annual Report, 1918-19. 1s. 3d.

Ministry of Health. The Vaccination Order, 1919. 1½d. Provisional Orders (No. 1). 1½d. Draft of Anatomy Acts, Transfer of Power Order, 1919.—1½d.

Nurses' Registration (No. 2) Bill. 3d.

National Relief Fund. Report on Administration to June 30, 1919. 2½d.

### Matrons' and Sisters' Appointments.

Booth Hall Infirmary, Blackley, Manchester.—Miss A. Ball has been appointed sister. She was trained at Tranmere Infirmary, Birkenhead, and since has been private nursing.

Deans Isolation Hospital, South Shields.—Miss L. E. Shaw has been appointed assistant matron. She was trained at the Royal Victoria Infirmary, Newcastle-on-Tyne, and North-Western Fever Hospital, Hampstead, and has been ward sister at Plaistow Fever Hospital, London, and on active service at home and abroad under the T.F.N.S.

Essex County Hospital, Colchester.—Miss Pewter has been appointed theatre sister. She was trained at the Royal Portsmouth Hospital.

Isle of Wight County Council.—Miss S. E. Bailey has been appointed county superintendent nurse. She was trained at the Infirmary, Keighley, Yorks, and since has been district nurse at Tywardreath; Queen's Nurse at Middlewich, Cheshire; and health visitor under the Urban Council, Middlewich.

Mutford and Lothlingland Union Infirmary, Oulton, near Lowestoft.—Miss L. E. Bayliss has been appointed superintendent nurse. She was trained at the Croydon Infirmary, and has held the appointments of superintendent nurse, Bromsgrove Infirmary; night superintendent, Harton Hospital, South Shields; head nurse, Ely Infirmary; and assistant superintendent, Stepney Infirmary.

The Royal Chest Hospital, City Road, E.C.—Miss D. Maguire has been appointed visiting sister. She was trained at Blackburn Royal Infirmary and at the Abingdon Fever Hospital. She has been tuberculosis nurse for Oxfordshire, has done private nursing, and holds the certificate of the Central Midwives Board.

### The College of Nursing (Limited by Guarantee).

EDINBURGH CENTRE.

An At Home was given at 29 Cantle Terrace by Miss White and Miss Miller (Q.V.J.I.N.) on Wednesday, November 26, to the members of the Edinburgh Centre of the College of Nursing. The guests, who numbered about 100, spent a delightful evening. In two rooms whist was played, while another was devoted to conversation and music. The violin solos by Miss Douglas were much appreciated, and Scottish and other songs were sung by some of the nurses. Later in the evening dancing was started, which was heartily taken advantage of and much enjoyed.

LIVERPOOL CENTRE.

Wednesday, December 10 (6.30 p.m.).—A members' meeting will be held in the lecture theatre, Royal Infirmary. After the meeting, at 7 p.m., C. J. Macalister, Esq., M.D., C.M., F.R.C.P., will lecture on "Diseases of Children."

### The Chadwick Trust.

The Chadwick Trustees have resolved, under the powers conferred upon them by the scheme which they administer, to award, in the year 1920, a Chadwick Gold Medal and a prize of £100 to the naval and military medical officer respectively in the British service who during the past five years of war shall be deemed to have distinguished himself the most in promoting the health of the men in the Navy and Army. The nomination to the Trustees for such presentation will, as provided by the Trust, be made by the Directors-General of the Naval and Military Medical Services respectively.

